# Image-Guided Peri-Tumoral Radiofrequency Hyperthermia-Enhanced Direct Chemo-Destruction of Hepatic Tumor Margins

**DOI:** 10.3389/fonc.2021.593996

**Published:** 2021-06-21

**Authors:** Minjiang Chen, Feng Zhang, Jingjing Song, Qiaoyou Weng, Peicheng Li, Qiang Li, Kun Qian, Hongxiu Ji, Sean Pietrini, Jiansong Ji, Xiaoming Yang

**Affiliations:** ^1^ Image-Guided Bio-Molecular Interventions Research & Division of Interventional Radiology, Department of Radiology, University of Washington School of Medicine, Seattle, WA, United States; ^2^ Key Laboratory of Imaging Diagnosis and Minimally Invasive Interventional Research of Zhejiang Province, Department of Radiology, Zhejiang University Lishui Hospital, Lishui, China; ^3^ Department of Pathology, Overlake Medical Center and Incyte Diagnostics, Bellevue, WA, United States

**Keywords:** radiofrequency hyperthermia, liver malignancies, chemotherapy, image-guided interventional oncology, peri-tumoral injection

## Abstract

**Purpose:**

To validate the feasibility of using peri-tumoral radiofrequency hyperthermia (RFH)-enhanced chemotherapy to obliterate hepatic tumor margins.

**Method and Materials:**

This study included *in vitro* experiments with VX2 tumor cells and *in vivo* validation experiments using rabbit models of liver VX2 tumors. Both *in vitro* and *in vivo* experiments received different treatments in four groups (n=6/group): (i) RFH-enhanced chemotherapy consisting of peri-tumoral injection of doxorubicin plus RFH at 42°C; (ii) RFH alone; (iii) doxorubicin alone; and (iv) saline. Therapeutic effect on cells was evaluated using different laboratory examinations. For *in vivo* experiments, orthotopic hepatic VX2 tumors in 24 rabbits were treated by using a multipolar radiofrequency ablation electrode, enabling simultaneous delivery of both doxorubicin and RFH within the tumor margins. Ultrasound imaging was used to follow tumor growth overtime, correlated with subsequent histopathological analysis.

**Results:**

In *in vitro* experiments, MTS assay demonstrated the lowest cell proliferation, and apoptosis analysis showed the highest apoptotic index with RFH-enhanced chemotherapy, compared with the other three groups (p<0.01). In *in vivo* experiments, ultrasound imaging detected the smallest relative tumor volume with RFH-enhanced chemotherapy (p<0.01). The TUNEL assay further confirmed the significantly increased apoptotic index and decreased cell proliferation in the RFH-enhanced therapy group (p<0.01).

**Conclusion:**

This study demonstrates that peri-tumoral RFH can specifically enhance the destruction of tumor margins in combination with peri-tumoral injection of a chemotherapeutic agent. This new interventional oncology technique may address the critical clinical problem of frequent marginal tumor recurrence/persistence following thermal ablation of large (>3 cm) hepatic cancers.

## Introduction

Advanced ablative and embolic techniques in interventional oncology have become important tools in the multidisciplinary approach to manage patients with primary and secondary liver malignancies. Image-guided percutaneous ablation techniques include ethanol ablation (EA), radiofrequency ablation (RFA), microwave ablation (MWA), cryoablation, and irreversible electrophoresis (IRE). Each technique has its own implicit advantages and disadvantages. EA is currently uncommon in clinical practice due to its high local tumor progression rate, which usually requires multiple treatment sessions (1, 2). Cryoablation of medium-to-large tumors often results in serious complications (particularly in the case of liver cirrhosis) and tumor recurrences (3, 4). To date, there is no sufficient data to support the superiority of IRE over the other ablation techniques (5, 6). Embolic (non-ablative) interventional techniques primarily include chemoembolization and radioembolization. The primary limitation of chemoembolization is the frequent development of tumor resistance to chemotherapy (7), while radioembolization is often technically complex (requiring two separate angiographic procedures for vascular mapping/lung-shunt fraction calculations and treatment infusion) with high cost and uncertain future for reimbursement by major insurance payers (8).

Both RFA and MWA are thermal ablation techniques. RFA—the older and more established technique—is universally adapted, well studied, and recommended by different organizations around the world. The use of MWA has been increasing in recent years due to the theoretical advantages of decreased heat sink effect and creation of wider and better regulated ablation zones compared with RFA (9, 10). However, due to the limitations in the achievable size and distribution of the ablation zone, thermal ablation—and indeed all ablative techniques—are most suitable for treating only small tumors (< 3 cm) (11, 12). In treating medium (3–5 cm) to large (5–7 cm) lesions, thermal ablation generally results in positive tumor margins (13, 14). The challenge of adequately ablating larger tumors is even greater in light of the fact that an effective and safe ablative zone must extend 1 cm beyond the tumor margin (so-called the surgical margin) using the current thermal ablation technology (15, 16). Several factors contribute to this phenomenon: 1) RFA thermal energy can be carried away by the neighboring vasculature at the tumor periphery (the so-called heat-sink effect); 2) intentional avoidance of MWA’s rapid over-heating by the operator in order to protect adjacent critical structures (such as vasculature, bile ducts, the gastrointestinal tract, and diaphragm); 3) infiltrative growth patterns of malignant tumors involving the formation of micro-satellite lesions or micro-venous tumor emboli beyond the surgical margin of ablated tumors; and 4) off-center positioning of the ablation electrode due to limited access windows (17, 18).

Several combination adjuvant and neoadjuvant approaches have been tested to overcome the above limitations (19). One example is to perform bland embolization or chemoembolization prior to RFA, with the intent of reducing downstream hepatic arterial blood flow and diminishing the “heat-sink” effect (20). However, this combination requires two consecutive treatment sessions, exposing patients to the risks of both procedures with increasing the possibility of multifocal tumor recurrence/progression through repeated manipulation (21). Another example of combination therapy is to administer systemic chemotherapy in addition to RFA. This involves the limitations of systemic chemotherapy, including possible sub-therapeutic drug dose reaching the tumor site, toxicity to other vital organs, and frequent development of chemo-resistance. A few reports describe a combination of RFA with radioembolization using Yttrium-90. This approach is not only expensive and technically cumbersome but also currently un-reimbursable by insurances (22). Thus, to date, there is no ideal imaging-guided interventional technique for efficiently eradicating all tumor cells in medium- and large-sized malignant tumors.

**Figure 1:**
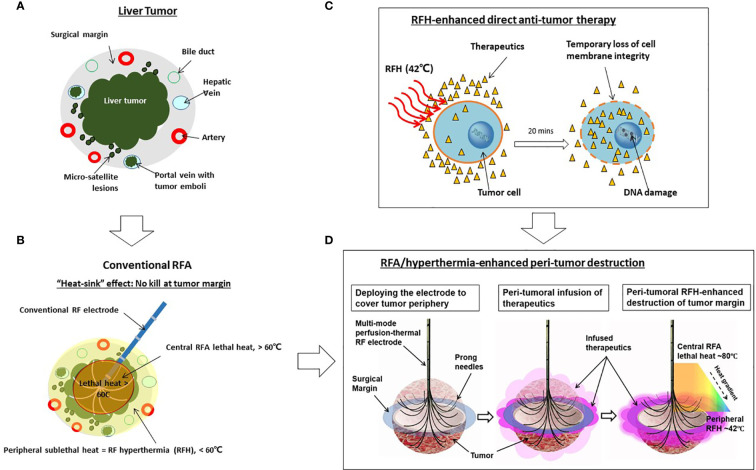
Development of the “one-stop-shop” interventional technique for locoregional treatment of medium-to-large liver tumors. **(A)** A cross-sectional view of a liver tumor with infiltrating border, micro-satellite lesions, and venous tumor emboli contained within the 1-cm surgical margin. **(B)** Conventional thermal ablation of medium-to-large tumor suffers from incomplete ablation at the tumor periphery. This is largely due to the “heat sink” effect associated with RFA, which results in sublethal hyperthermia (< 60°C) at the periphery of tumors. **(C)** The mechanism of RFH-enhanced anti-tumor therapy. Therapeutic agents, introduced into the tumor *via* either percutaneous needle injection or intraarterial catheter infusion, dwell primarily in the extracellular space with very limited intracellular uptake. Applying 42°C-RFH for 20 mins facilitates a temporary loss of cell membrane integrity, allowing therapeutic agents to enter cells and exert cytotoxic effects more readily. **(D)** Our strategy of the “one-stop-shop” solution to overcoming incomplete ablation of medium-to-large liver tumors. The multimodal perfusion thermal electrode permits (i) creation of lethal RFA heat (60-100°C) for necrotic ablation at the tumor center; (ii) direct infusion of therapeutics into the tumor periphery (surgical margin), and (iii) maintenance of a stable sublethal peritumoral hyperthermia (~42°C), which enhances destruction of tumor margins by increasing the uptake and cytotoxicity of therapeutic agents.

To address this critical clinical problem, we propose percutaneously deliver anti-tumor agents to the tumor periphery simultaneously during image-guided ablation sessions. Recently, studies from our group and others also have confirmed that RFA-mediated hyperthermia (RFH) can greatly enhance local gene/chemo/oncolytic therapies of various malignancies, including hepatic tumors (23–26). The groundwork laid by these studies has allowed us to develop an “one-stop shop” interventional technique by which medium-to-large liver tumors can be treated simultaneously with both site-directed chemotherapy and RFA (**Figure 1**).

## Materials and Methods

### Study Design

This study included two components: (a) *in vitro* experiments to prove the principle of the new concept, “RFH enhancement of the tumor-killing effect of chemotherapeutic agents” on VX2 tumor cells; and (b) *in vivo* experiments to validate the feasibility of this new interventional oncologic technique, “Imaging-guided RFH-enhanced direct chemo-destruction of tumor margin tissues”, in rabbit models with orthotopic VX2 liver tumors using a multi-modal perfusion-thermal RFA electrode.

### *In Vitro* Experiments

#### RFH-Enhanced Killing Effect of Chemotherapeutic on VX2 Tumor Cells

VX2 tumor cells (IDAC, Tohoku University, Japan) were seeded (8 × 10^4^ per well) in four chamber cell culture plates (Thermo Fisher Scientific, Rochester, NH). RFH was performed by attaching a custom-made 0.022-inch radiofrequency heating wire to the bottom of the chamber and connecting it to a radiofrequency generator. A sterilized 1.1-mm fiberoptic temperature probe was placed in the bottom of each chamber and connected to a thermometer (PhotonControl, Burnaby, British Columbia, Canada) for real-time monitoring of the temperatures during the RFH.

The cells were divided into four groups (n=6/group), which were treated by (a) combination therapy consisting of doxorubicin (Doxo, 13.9 μM) with RFH at 42 ± 1°C for 20 min; (b) RFH alone; (c) Doxo alone; and (d) phosphate-buffered saline (PBS). The dose of Doxo (Mylan Institutional LLC, Rockford, IL) for cell treatment was determined by its 50% inhibitory concentration (IC50).

### Cell Viability Assay

Cell viability was evaluated by MTS [3-(4,5-dimethylthiazol-2-yl)-5-(3-carboxy-methoxyphenyl)-2-(4-sulfophenyl)-2H-tetrazoliu] assay (Promega Corporation, Madison, WI), and measured at 490 nm with a microplate reader (VersaMax; Molecular Devices, Sunnyvale, Calif.). Relative cell viability of the four groups was calculated using the equation of A_treated_ − A_blank_/A_control_ − A_blank_, where A is the absorbance of formazan. Then, cells culture slides were fixed in 4% paraformaldehyde, counterstained with 4’,6-diamidino-2-phenylindole (DAPI; Vector Laboratories, Burlingame, Calif.), and then imaged with a fluorescence microscope.

### Apoptosis Measurement

The percentages of cell apoptosis were quantified by Annexin V/Propidium iodide (PI) dual staining kit (BD Biosciences, San Diego, CA). Samples were analyzed by a FACScan flow cytometer (BD Biosciences). Data were analyzed using the FlowJo software version 10 (FloJo Data Analysis Software, Ashland, Ore).

### *In Vivo* Experiments

The experimental protocol was approved by our Institutional Animal Care and Use Committee. Twenty-four female New Zealand white rabbits (2.5–3.5 kg) were used for the *in vivo* experiments. All experiments involving rabbits were performed under general anesthesia with the inhalation of isoflurane delivered in oxygen at a flowrate of 1 L per minute.

### Creation of Liver VX2 Tumors in Rabbits

VX2 tumor cells were implanted into the thigh muscles of donor rabbits. Approximately 2 weeks later, the donor rabbits were euthanized with intravenous injection of overdose pentobarbital at 100 mg/kg body weight. Then, VX2 tumors in the thigh muscle were harvested and was minced into 1 mm^3^ pieces under sterile conditions.

**Figure 2:**
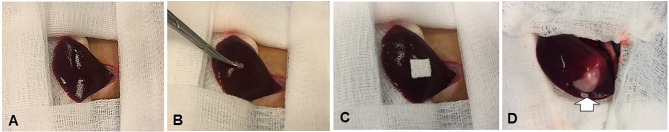
Creation of the New Zealand white rabbit models with orthotopic liver VX2 tumors. An approximately 1-mm^3^ minced VX2 tumor is directly implanted into the subcapsular area of the left lobe of the liver by ophthalmic bend tweezers **(A, B)**, followed by 5-min manual compression of the tumor injection site with a gelatin sponge **(C)**. Two weeks later, the tumors are created [arrow on **(D)**].

For the recipient rabbits, under sterilization a 3- to 40-cm incision was made along the midline below the subxiphoid process to expose the left liver lobe (**Figure 2**). A 1-mm^3^ minced tissue fragment from the donor VX2 tumor was directly implanted into the subcapsular parenchyma of the left liver lobe, followed by 5-min manual compression of the tumor injection site with a gelatin sponge (Pharmacia & Upjohn Co, Kalamazoo, MI), and the closure of the abdominal incision with layered sutures.

### Introtumoral RFA-Induced Hyperthermia to Enhance Peri-Tumoral Chemo-Destruction of VX2 Liver Tumor

**Figure 3:**
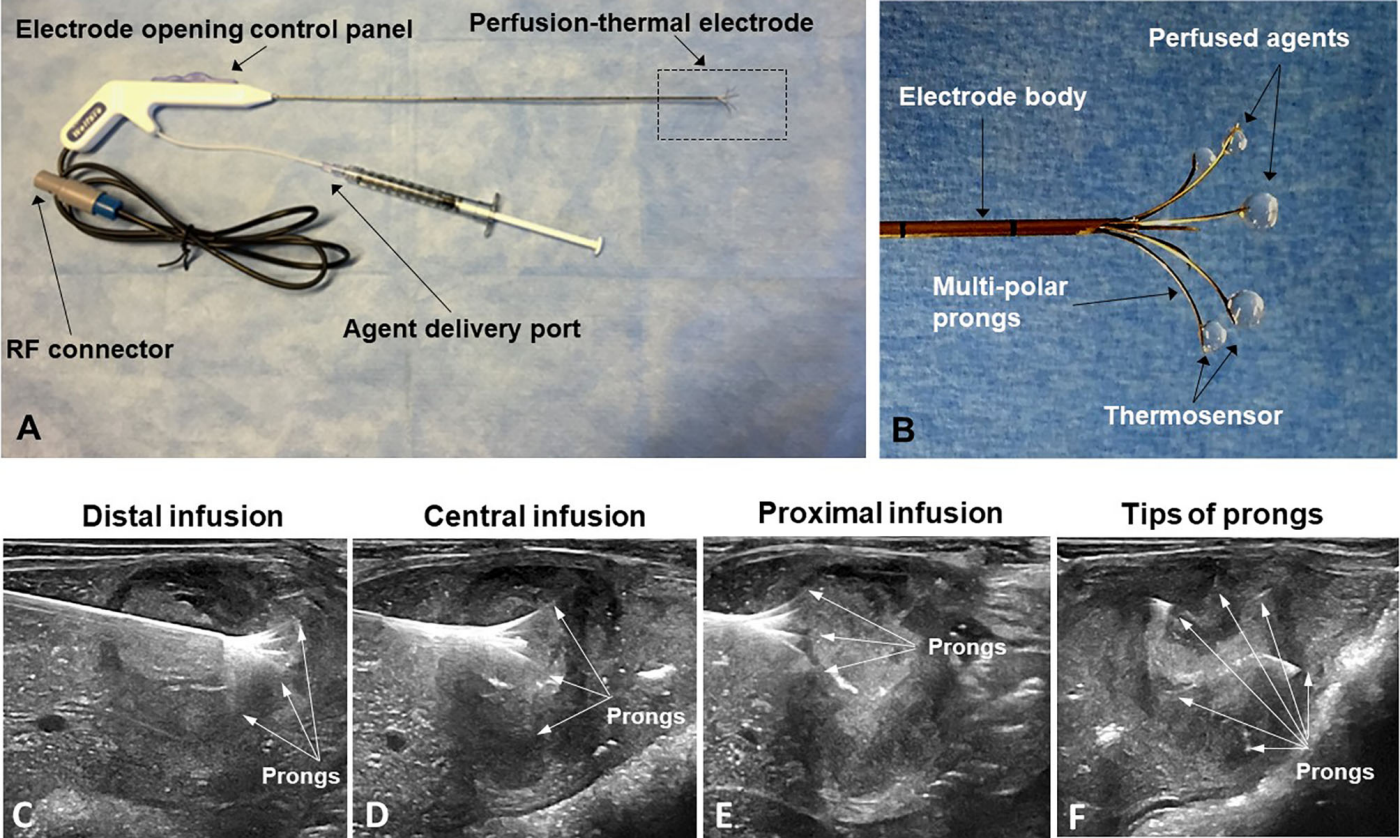
**(A, B)** The multimodal perfusion thermal electrode with multipolar agent infusion prongs. Micro-thermosensors are equipped at the tips of the prongs for real-time measurement of peritumoral RFH at approximately 42°C, while the central-tumoral RFA tempreature from the electrode body was pre-set at approximately 80°C. **(C–F)** The stepwise peri-tumor delivery of doxorubicin under real-time ultrasound guidance. The perfusion-thermal electrode is advanced, sequentially into the portion of the tumor most distal from the entry site. At regular intervals during electrode advancement through the tumor, the electrode prongs are opened to the desired size array, with the objective of completely enveloping the tumor peripheral zone for sufficient delivery of the chemotherapeutic agent. During delivery of the chemotherapeutic agent, RFA-mediated RFH at the tumor margin is generated with the same device to further enhance chemo-destruction of tissues at the tumor margins. **(A–C)** Longitudinal sonographic images; **(D)** Transverse image.

The peri-tumoral infusion of Doxo was performed using a multi-modal perfusion-thermal RFA electrode system, which had multipolar prongs enabling simultaneous peri-tumoral delivery of chemotherapeutic agents and thermal ablation (**Figure 3**). The array sizes of the prongs were adjustable at a range of 1 to 5 cm in diameter. Peri-tumoral sub-lethal RFH (approximately 42°C) was induced by the temperature gradient from the central-tumoral lethal RFH (approximately 80°C). Simultaneously, a chemotherapeutic agent was delivered to the margins of ablated tumors (**Figure 3**).

Twenty-four rabbits were divided into four treatment groups (n=6/group): (a) combination therapy consisting of peri-tumoral infusion of Doxo (4 mg/kg) plus RFA-induced peri-tumoral RFH (RFA/RFH) at 42 to 44°C for 20 min; (b) RFA/RFH alone; (c) Doxo alone; and (d) saline.

We used a three-step procedure to cover the entire tumor margin. Under ultrasound imaging guidance (Sonosite Inc, Bothel, WA), we first positioned the tip of the multi-modal electrode in the distal portion of the VX2 tumor, then opened the electrode prongs to completely cover the tumor margin. While infusing 4 mg/kg Doxo into the tumor periphery under ultrasound guidance, RFA/RFH was generated at 42°C to 44°C for 20 min and monitored/controlled using the digital panel of the electrode system. Then, the electrode was withdrawn to the central portion of the tumor, and peri-tumoral infusion of Doxo with simultaneous RFA/RFH again for 20 min was again performed. Finally, the proximal portion of the tumor was treated in the same manner (**Figure 3**).

### Post-Treatment Follow-Up

Ultrasound imaging was performed to assess the changes in tumor size at days 0, 7, and 14 after the treatments. The transverse (X), longitudinal (Y), and AP (Z) diameters of the tumors were measured on the ultrasound images. The volume of each tumor was calculated according to the equation: V= X*Y*Z*π/6, where V is tumor volume. Data were presented as relative tumor volume (RTV) by using the following equation: RTV = V_Dn_/V_D0_, where Dn represents days after treatments, D0 is the day before treatments, and Dn is the day after the treatments.

### Histopathology

Tumor tissues were harvested, fixed in 4% paraformaldehyde solution, and embedded in paraffin. These tumor tissues were cut into 5-mm sections for the following histopathological staining preparations: (a) hematoxylin-eosin (HE) to confirm the formation of liver VX2 tumor; (b) terminal deoxynucleotidyl transferase biotin-dUPT nick end labeling (TUNEL) to determine tumor cell apoptosis; and (c) Ki-67 immunostaining (Abcam, Cambridge, MA) to assess tumor proliferations among four groups. Positive cells were imaged using a microscope (200× amplification; Olympus, Tokyo, Japan), and counted by Image-pro Plus 6.0 software (Media Cybernetics, Silver Spring, MD).

The systemic toxicity of Doxo in different groups was also evaluated by hematoxylin-eosin (H&E) staining, which was performed on the lung, heart, kidney, and spleen.

### Statistical Analysis

Statistical software (SPSS, Version 19.0; Chicago, Ill) was used for all data analyses. The non-parametric Mann-Whitney U test was used to compare (i) relative proliferation rates among different cell groups, (ii) tumor volumes, (iii) proliferation rates, and (iv) apoptotic indexes at different time points among different animal groups with various treatments. Statistical significant was assumed for *P*<0.05.

## Results

### *In Vitro* Confirmation: RFH-Enhanced Doxo Killing Effect on VX2 Cells

**Figure 4:**
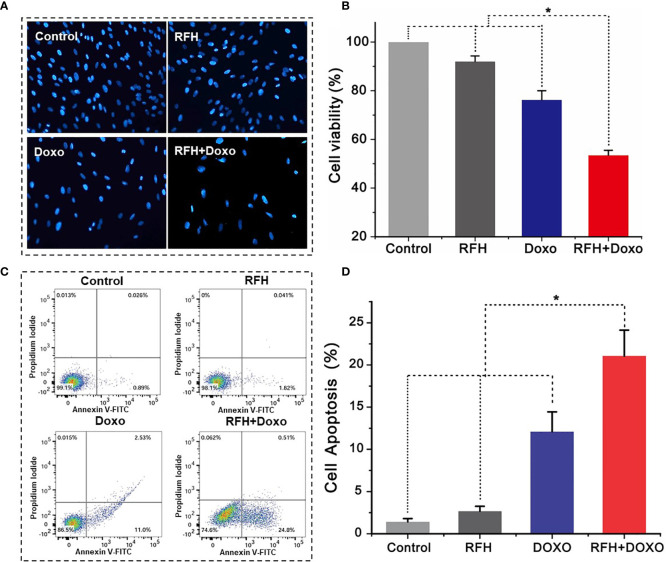
*In vitro* experiments on therapeutic effect among different treatments groups. **(A)** Microscopy shows an obvious decrease of viable cells in the RFH + Doxo group, compared to other control groups. **(B)** MTS assay further confirms that RFH-enhanced chemotherapy results in the lowest cell viability, compared to other control treatments [Doxo+RFH (53.47 ± 2.01%) *vs* Doxo (76.23 ± 3.80%), RFH (92.03 ± 2.24%), and saline (100%); P<0.05, n=6 per group]. **(C)** The combination therapy group displays the highest percentage of apoptotic cells. **(D)** Quantitative analysis of flow cytometry, demonstrating significant increase in apoptotic cells with the combination therapy, compared to other treatments [Doxo+RFH (21.8 ± 3.05%) *vs* Doxo (12.10 ± 2.34%), RFH (2.67 ± 0.59%), and saline (1.43 ± 0.37%); P<0.05, n=6 per group]. *p < 0.05.

Confocal microscopy showed the lowest survival of cells in the combination therapy group (RFH+Doxo), compared with the other three treatments groups (**Figure 4A**). MTS assay further confirmed the significantly lower cell viability with combination therapy compared with Doxo alone, RFH alone, and saline (53.47 ± 2.01% *vs* 76.23 ± 3.80% *vs* 92.03 ± 2.24% vs 100%, respectively; *P*<0.01) (**Figure 4B**). Flow cytometry displayed the highest percentage of apoptotic VX2 cells in the combination therapy group compared with the other three groups (21.8 ± 3.05% *vs* 12.10 ± 2.34% *vs* 2.67 ± 0.59% *vs* 1.43 ± 0.37%, respectively; *P*<0.01) (**Figures 4C, D**).

### *In Vivo* Validation: RFA/RFH-Enhanced Peri-Tumoral Chemo-Destruction of VX2 Liver Tumors

**Figure 5:**
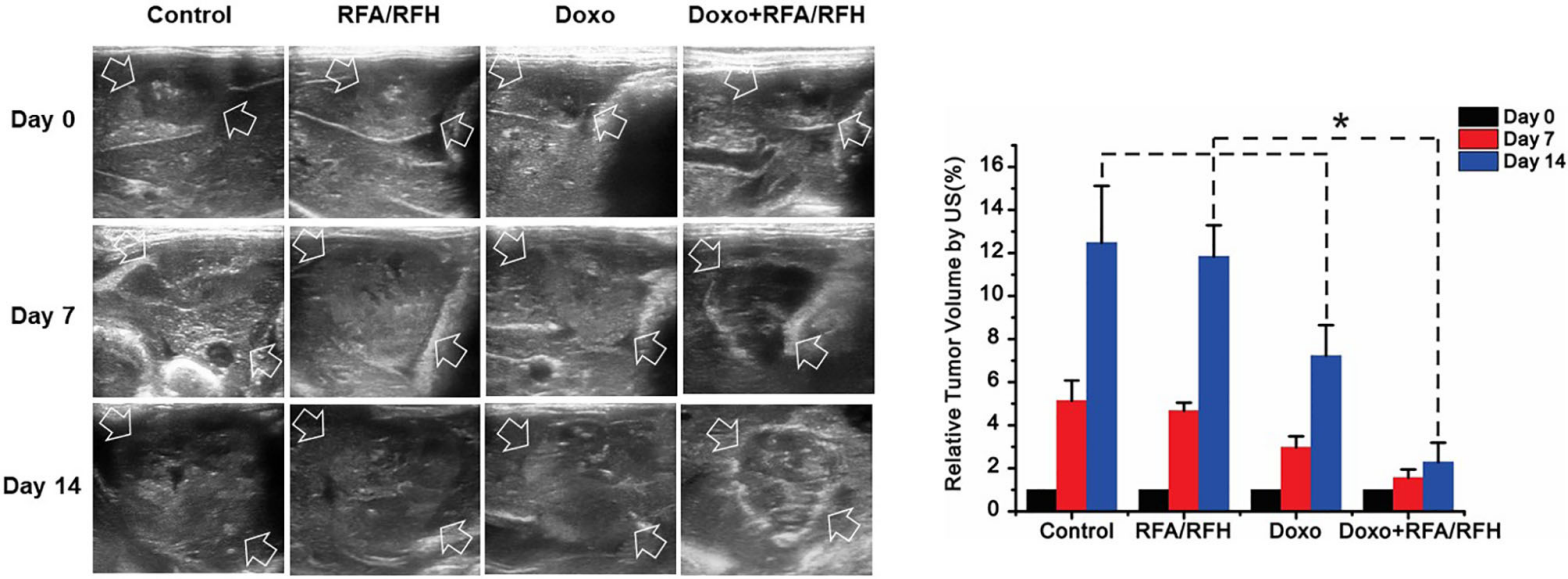
*In vivo* studies using rabbit models with orthotopic liver VX2 tumors in four groups with different treatments to validate the feasibility of this new interventional oncologic technique. (Left) Ultrasound imaging demonstrates a significant decrease of tumor growth rate at day 14 post-treatment with RFH-enhanced therapy, compared to the other three control treatments [Doxo+RFA/RFH (2.29 ± 0.9) *vs* Doxo (7.23 ± 1.43), RFA/RFH (11.84 ± 1.45), and saline (12.48 ± 2.64); P<0.05, n=6 per group, Right]. *P < 0.05.

**Figure 6:**
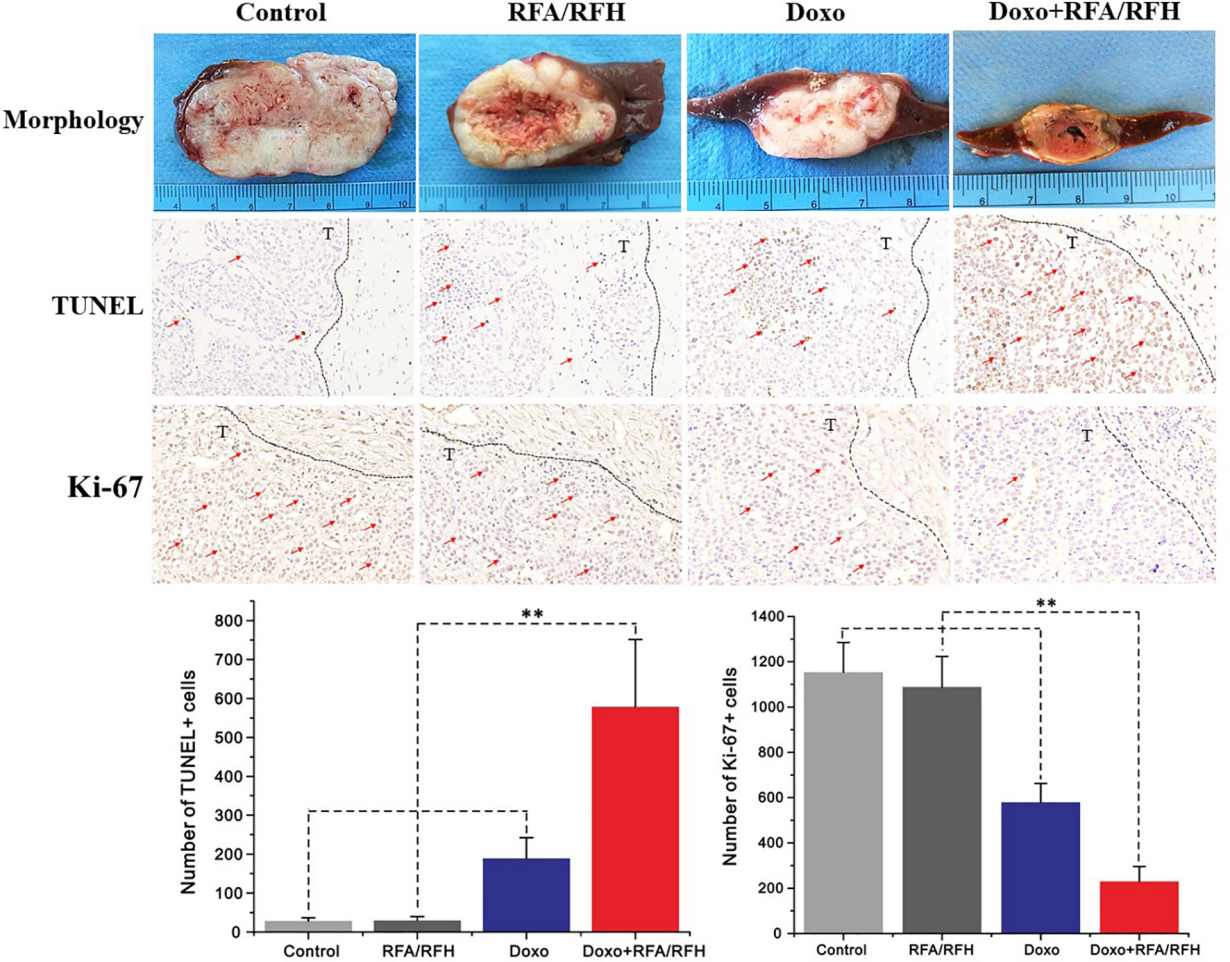
The gross pathology and the corresponding immunohistochemical staining of rabbit orthotopic tumor tissues after the different treatments. The morphology shows the smallest tumor size in the combination therapy group compared to the other three groups. TUNEL staining for apoptosis further confirms an significantly enhanced tumoricidal effect of the combination therapy, and the largest number of brown-stained cells could be detected in the combination treatment, compare to other treatments [Doxo+RFA/RFH (578.67 ± 173.01) *vs* Doxo (189.33 ± 9.95), RFA/RFH (29.83 ± 9.95), and saline (28.17 ± 8.73); n=6 per group, P<0.01]. Ki-67 staining shows a significant inhibition of cancer cell proliferation in the combination therapy group, as evidenced by the fewest number of brown-stained cells [Doxo+RFA/RFH (230.17 ± 66.08) *vs* Doxo (580.33 ± 82.20), RFA/RFH (1089.00 ± 134.50), and saline (1154.00 ± 131.31); n=6 per group, P < 0.01]. **P < 0.01.

Ultrasound imaging showed the smallest relative tumor volume in the combination therapy, compared with Doxo alone, RFA/RFH alone, and saline (2.29 ± 0.9 *vs* 7.23 ± 1.43 *vs* 11.84 ± 1.45 *vs* 12.48 ± 2.64, respectively, *P*<0.01) (**Figures 5**, **6**). TUNEL staining demonstrated the significantly higher apoptosis in combination therapy (578.67 ± 173.01 *vs* 189.33 ± 9.95 *vs* 29.83 ± 9.95 *vs* 28.17 ± 8.73, *P*<0.01) (**Figure 6**). Correspondingly, the cell proliferation analysis by Ki-67 immunostaining further displayed the lower proliferation activity in the combination therapy group, compared with the other three animal groups (230.17 ± 66.08 vs 580.33 ± 82.20 *vs* 1089.00 ± 134.50 *vs* 1154.00 ± 131.31, *P*<0.01) (**Figure 6**).

**Figure 7:**
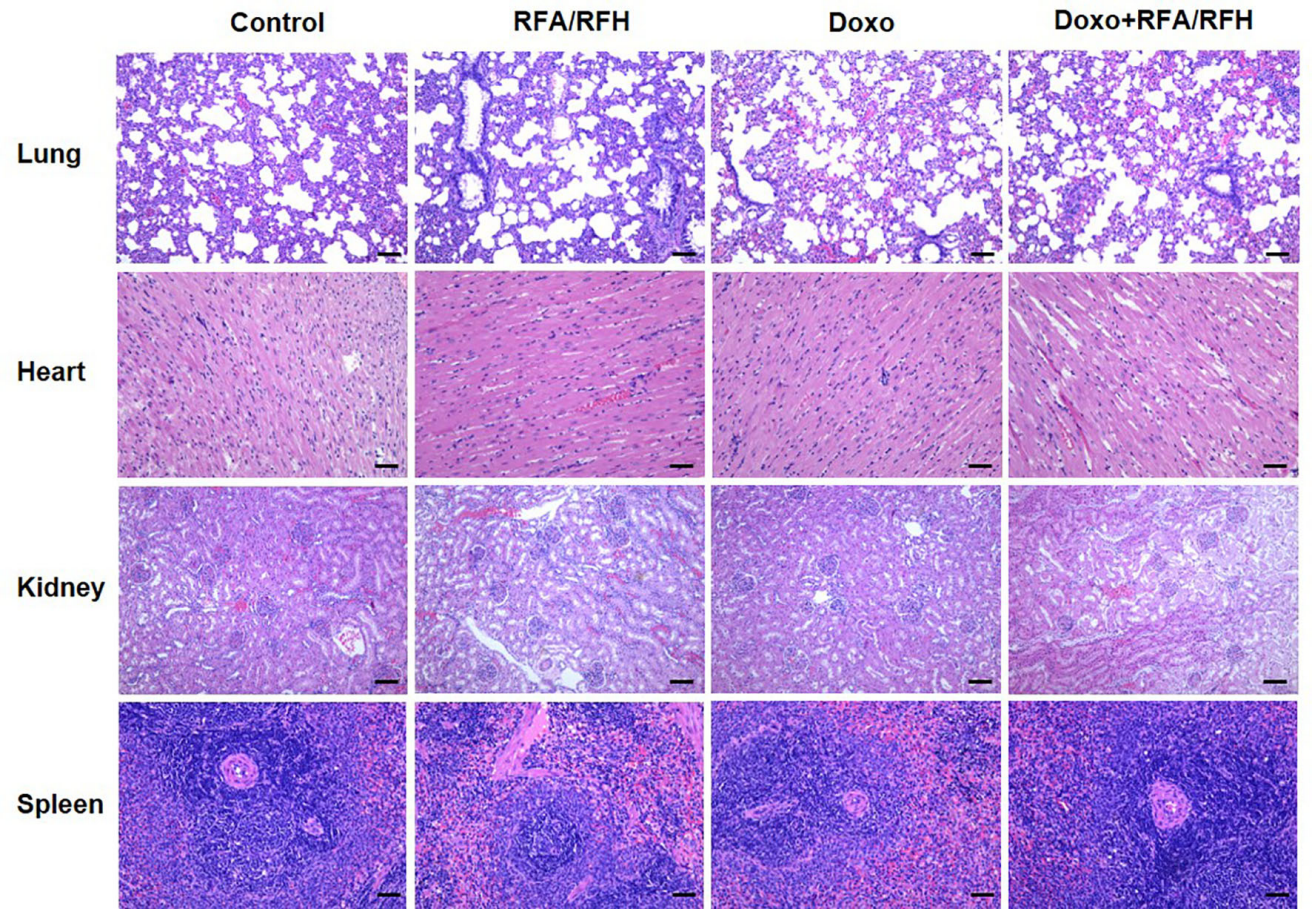
Representative major organ tissue (lung, heart, kidney, and spleen) stained with H&E 2 week after treatment. Scale bar represents 100 μm.

To further clarify the systemic toxicity of Doxo under this novel treatment mode, H&E staining was performed on the lung, heart, spleen, and kidney (**Figure 7**). The results of pathology show no abnormal morphological changes and differences among various study groups, suggesting that the local injection of DOXO did not produce systemic toxicity, and the treatment mode had good biosafety.

## Discussion

Hepatic malignancy (including hepatocellular carcinoma and hepatic metastases) is one of the most common and lethal cancers worldwide (27). Surgical resection is the gold standard curative treatment for liver malignancies. However, only 10% of patients are eligible for surgical resection at the time of tumor diagnosis, due to extent of disease, compromised liver function, and additional comorbidities, including cardiovascular and pulmonary disease (28).

Imaging-guided thermal ablation techniques, primarily including RFA and MWA, have become effective curative locoregional treatment modalities for small-sized hepatic tumors (<3 cm) (29, 30). However, the incidence of tumor recurrence originating at the margin of treated tumor (or within 1 cm of the tumor’s surgical margin) is high when ablating medium (3–5 cm) and large (5–7 cm) hepatic lesions (31–33). In the current study, we attempted to overcome this critical clinical dilemma by developing a new interventional technique using peri-tumoral RFH induced by RFA, which can enhance the destruction of residual tumor cells by a first-line chemotherapeutic agent, doxorubicin (Doxo), at the margin of hepatic tumors.

The results of *in vitro* experiments in our study demonstrated the enhanced tumor cell killing effect of the combination treatment of RFH plus chemotherapy, manifesting as significantly higher number of apoptotic cells and lower cell viability compared with the other treatment groups. *In vivo* experiments on rabbit models with orthotopic liver tumors further confirmed that the tumor growth was remarkably retarded in the group with the combination therapy compared with the other groups, as revealed by decreased tumor volume, increased apoptosis, and decreased cell proliferation. Our study provides evidence of (a) improved chemotherapeutic efficacy at the tumor margin due to RFA-induced peri-tumoral hyperthermia; and (b) the advantages of the multi-modal perfusion RF ablation electrode system, which has multiple functions of peri-tumoral delivery of high-dose chemotherapeutic agents, generation of RF thermal energy, and real-time temperature monitoring.

This technical development may provide a unique strategy to eliminate the tumor cells at or near the surgical margins of ablated tumors, which are difficult to treat in medium and large-sized tumors by current thermal ablation techniques (34). To ensure full chemotherapeutic coverage of the tumor margins, doxorubicin was infused at the distal, central and proximal portions of the tumor using the same thermal electrode under ultrasound imaging guidance.

We followed up the tumor growth post-treatments only for up to 14 days, due to the consideration that a longer follow-up period would result in tumor size exceeding ten percent of body weight in the animal test subjects, which was not be allowed by our Institutional Animal Care and Use Policy. Since this study primarily focused on the development of our technique, we did not explore the potential mechanisms of RFH-enhanced chemotherapy. We can conjecture that these might involve increasing permeability of cell membranes, increasing cellular metabolism, and accelerated apoptosis of tumor cells by hyperthermia (11, 35, 36).

## Conclusion

This study has validated the feasibility of using central-tumoral RFA-induced RFH to enhance the effectiveness of peri-tumoral chemo-destruction of tumor margins. We believe that this study may facilitate the development of a new “one-stop-shop” interventional oncology technique, which can enhance the treatment adequacy and address the high rate of recurrence associated with RFA of liver tumors larger than 3 cm.

## Data Availability Statement

The original contributions presented in the study are included in the article/supplementary material. Further inquiries can be directed to the corresponding authors.

## Ethics Statement

The animal study was reviewed and approved by Institutional Animal Care and Use Committee of University of Washington.

## Author Contributions

XY and JJ developed the concept and designed the experiments. MC, FZ, JS, and QW performed the experiments and wrote the manuscript. PL, QL, KQ, and HJ collected and analyzed data. SP revised the manuscript. All authors contributed to the article and approved the submitted version.

## Funding

This work was supported by the NIH RO1EBO12467 and NIH R01EB028095 grants, the National Key Research and Development projects Intergovernmental Cooperation in Science and Technology of China (no. 2018YFE0126900), the Key Research and Development Project of Zhejiang Province (no. 2018C0302), and Key Program of National Natural Science Foundation of China (no. 81430040).

## Conflict of Interest

The authors declare that the research was conducted in the absence of any commercial or financial relationships that could be construed as a potential conflict of interest.

## Abbreviations

RFH, Radiofrequency hyperthermia; RFA, radiofrequency ablation; HCC, hepatocellular carcinoma; EA, ethanol ablation; IRE, Irreversible electrophoresis; Doxo, doxorubicin; PBS, phosphate-buffered saline; RTV, relative tumor volume; HE, hematoxylin and eosin; PI, propidium iodide; RS, radiation segmentectomy; TACE, transarterial chemoembolization; MWA, microwave ablation.
